# Comparison of knee motion on Earth and in space: an observational study

**DOI:** 10.1186/1743-0003-3-8

**Published:** 2006-04-13

**Authors:** Mark C Pierre, Kerim O Genc, Micah Litow, Brad Humphreys, Andrea J Rice, Christian C Maender, Peter R Cavanagh

**Affiliations:** 1 Department of Biomedical Engineering, Lerner Research Institute, Cleveland Clinic, Cleveland, OH, USA; 2 Center for Space Medicine, Cleveland Clinic, Cleveland, OH, USA; 3 Department of Orthopaedic Surgery, Cleveland Clinic, Cleveland, OH, USA; 4 Orthopaedic Research Center, Cleveland Clinic, Cleveland, OH, USA; 5 Case Western Reserve University, Cleveland, OH, USA; 6 ZIN Technologies, Inc., Brook Park, OH, USA; 7 NASA-Johnson Space Center, Houston, TX, USA

## Abstract

**Background:**

Spaceflight has been shown to cause atrophy, reduced functional capacity, and increased fatigue in lower-limb skeletal muscles. The mechanisms of these losses are not fully understood but are thought to result, in part, from alteration in muscle usage.

**Methods:**

Knee-joint angles and lower-extremity muscle activity were measured continually, via elecrogoniometry and surface electromyography respectively, from two subjects during entire working days of activity on Earth and onboard the International Space Station (ISS).

**Results:**

On Earth the distribution of angular positions of the knee was typically bimodal, with peaks of >75 degrees of flexion and in almost full extension (<15 degrees of flexion). However, on the ISS, a single peak in the mid-range of the available range of motion was seen. The knee joint was also moved through fewer excursions and the excursions were smaller in amplitude, resulting in a reduced span of angles traversed. The velocities of the excursions in space were lower than those used on Earth.

**Conclusion:**

These results demonstrate that, in space, overall knee-joint motion is reduced, and there is a transformation in the type of muscle action compared to that seen on Earth, with more isometric action at the expense of concentric and particularly eccentric action.

## Background

Spaceflight has been shown to cause atrophy, reduced functional capacity, and increased fatigue in skeletal muscles of the lower limbs, with the greatest change observed in "anti-gravity" muscles, primarily the leg extensors [1-5]. The mechanisms of these losses are not fully understood but can be attributed in part to altered gene expression of myofibril proteins [6,7] which is closely related to muscle usage [8]. One of the primary functions of skeletal muscle, as demonstrated by the leg extensors, is to routinely develop forces against gravity. Active and passive tensions have been shown to be essential for myofibril hypertrophy [9,10] and the reductions of either tension during spaceflight most likely contribute to the muscle atrophy and functional losses observed [11].

Joint angles can be good indicators of muscle length if combined with an appropriate mathematical model of the joint [12]. Even without such a model, inferences about the relative lengths of joint muscles can be made. A tendency for the knee to remain in a somewhat flexed position during activities in space has been previously reported [13,14] and this implies that the muscles crossing the knee joint experience altered patterns of usage. Therefore, the objective of this report is to document knee-joint motion in the same subjects both onboard the International Space Station (ISS) and on Earth.

## Methods

Angles of the knee joint and muscle activity of the vastus medialis (VM) and biceps femoris (BF) were measured continually during entire working days of activity (approximately 8 hours) in the same subjects on Earth and onboard the ISS. The Institutional Review Boards of the Cleveland Clinic Foundation (Cleveland, OH), and NASA's Johnson Space Center (Houston, TX) approved the protocol in advance and subjects provided written informed consent before participating in the experiment. A custom-built Lower Extremity Monitoring Suit (LEMS), with incorporated electrogoniometers (Biometrics, Ltd., Cwmfelinfach, UK) and surface electrodes, recorded the knee-joint angles and muscle activity on a wearable computer, allowing crewmembers to move freely and untethered. The electrogoniometers were attached by secure Velcro anchors to the lateral side of the right knee and were calibrated to a 1 *g*-like standing position (full extension) at the start of each collection. Two crewmembers participated in this study (Subject 1: 45 yrs, 80 kg, 1.7 m pre-flight; Subject 2: 46 yrs, 75 kg, 1.8 m pre-flight). Subject 1 collected data for 4 typical working days (8.4 ± 0.6 hrs) on Earth and 6 days (9.7 ± 0.4 hrs) onboard the ISS. Subject 2 collected data for 3 days (7.3 ± 0.1 hrs) on Earth and 4 days (7.2 ± 0.8 hrs) onboard the ISS.

**Figure 1:**
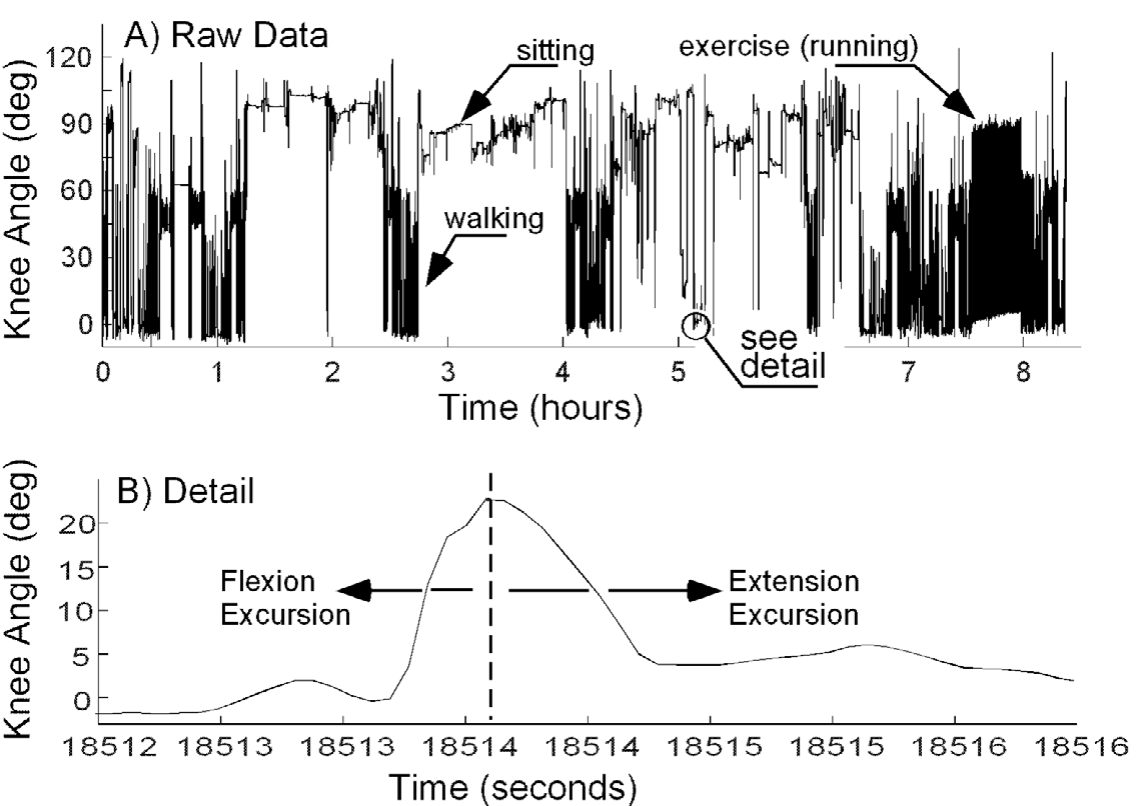
Data from a typical experimental trial. (A) Knee angle recorded for an entire day of activity on Earth for Subject 1. Specific sections depicting walking, running, and sitting are indicated. Zero degrees indicates full knee extension. (B) Detailed view of a 4s section of the above data demonstrating an excursion event. A transition from an extension excursion to flexion was detected when the change in angular direction exceeded a 3° fluctuation.

The angle of the knee joint was sampled continuously at 128 Hz throughout each working day. A knee angle of 0°, as sampled during standing, was defined as full extension. From these data, three parameters were calculated for the entire dataset: 1) the angular position of the knee, rounded to the nearest degree, at each sampling point; 2) the amplitude and direction (flexion or extension) of all excursions of >3° (Figure 1B); and 3) the average velocity of each excursion. Typical data for an entire working day on Earth are presented in Figure 1A. To account for different total sampling times, the number of occurrences of each joint angle was divided by the duration of the data collection in hours. The excursion was determined by measuring the amplitude of continuous motion in one direction, thus representing a monotonically increasing (extension) or decreasing (flexion) knee angle, as demonstrated in Figure 1B. A transition between flexion and extension excursions was indicated by a change in the direction of motion of >3°, thus discounting small fluctuations in knee angle. The excursions were grouped into 1-degree bins, and the number of occurrences at each excursion was normalized by the duration of the data in hours. The velocity of the excursions was linearly approximated by dividing the magnitude of each excursion by the total time for that excursion.

Muscle activity, collected by surface electromyography (EMG), was sampled at 1024 Hz. EMG data were cleaned using a 2000^th^-order band-pass finite impulse response zero-phase distortion filter (a 1000^th^-order finite impulse response filter was used with the data being first passed forward and then in reverse; see MATLAB's filtfilt function (Mathworks, Natick, MA, USA). The band-pass spectrum of the filter was from 20 to 400 Hz. The EMG data were enveloped by calculating an interval root mean square (RMS) over a period of 1/64 of a second. Using the interval RMS data from the resting calibration period, the mean of the RMS and the standard deviation of the RMS were calculated. The threshold value was then calculated to be the 95% confidence interval of the interval RMS. This methodology essentially creates a maximum enveloped RMS value during the threshold period. The muscle is then considered to be active when the interval RMS is greater than the threshold. Any muscle activation shorter than 0.150 seconds was not considered.

These data were then correlated with concurrent joint activity to determine whether the muscles of interest, VM and BF, were acting concentrically (during knee extension [VM] or flexion [BF]), eccentrically (during knee flexion [VM] or extension [BF]), or isometrically (during periods when the knee-joint angle did not change by more than 3° and the muscle was considered active). This characterization does not account for any differential length changes of the passive and active elements in muscle; rather it describes the length change of the muscle-tendon unit.

## Results

### Angular position of knee joint

**Figure 2:**
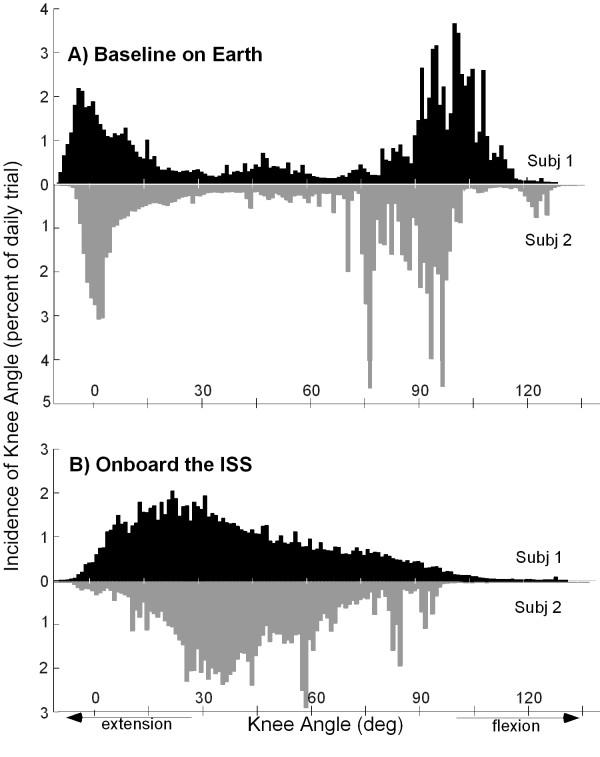
Typical histograms of the instantaneous angular position of the knee joint. Data for both subjects are shown (A) on Earth and (B) onboard the International Space Station (ISS). On Earth 73.0 ± 11.5% of the instantaneous knee angles occurred <15° and >75° ; onboard the ISS 74.6 ± 8.9% occurred within 15–75°).

Figure 2 shows typical histograms of the instantaneous angular position of the knee joint for both subjects during typical days on Earth (Figure 2A) and onboard the ISS (Figure 2B). A summary of data from all trials is presented in Table 1.

**Table 1:** Summary of results for angular position, excursion, and velocity.

	**Onboard the ISS**	**Earth**
**Mean Angular Position (deg)**		
Subj 1	36.9 ± 9.2	53.4 ± 13.1
Subj 2	48.2 ± 4.8	65.0 ± 6.2
		
**Modal Angular Position (deg)**		
Subj 1	23.7 ± 9.0	86.0 ± 17.7
Subj 2	40.8 ± 13.4	89.0 ± 10.5
		
**Total Number of Excursions per Hour**		
Subj 1	1.35 × 10^3^± 0.18 × 10^3^	2.66 × 10^3^ ± 0.26 × 10^3^
Subj 2	1.28 × 10^3^ ± 0.24 × 10^3^	1.94 × 10^3^ ± 0.44 × 10^3^
		
**Total Excursion Magnitude per Hour (deg)**		
Subj 1	3.7 × 10^4^ ± 0.6 × 10^4^	10.0 × 10^4^ ± 0.6 × 10^4^
Subj 2	3.1 × 10^4^ ± 0.5 × 10^4^	8.0 × 10^4^ ± 3.0 × 10^4^
		
**Mean Excursion Velocity (deg/sec)**		
Subj 1	65.7 ± 8.0	156 ± 13.9
Subj 2	63.0 ± 12.2	129 ± 55.2

Note: Standard deviations are for multiple collections under the same conditions.

The data collected on Earth shows a characteristic bimodal distribution, with peaks at full extension and at approximately 90° of flexion, whereas the data from onboard the ISS show a predominantly unimodal distribution, with a peak at approximately 30°-50° of flexion. In space, 74.6 ± 8.9% of the knee-joint angles were between 15° and 75°; on Earth, 73.0 ± 11.5% of the knee-joint angles were either <15° or >75°, when averaged across all days for both subjects. The mean knee-joint angle onboard the ISS, averaged across all trials and both subjects, was 41.4 ± 9.4°, with no statistical difference between the individual subjects (*p *= 0.05). The average modal knee-joint angle across all trials onboard the ISS was 30.5 ± 13.5°.

### Excursions of knee joint

**Figure 3:**
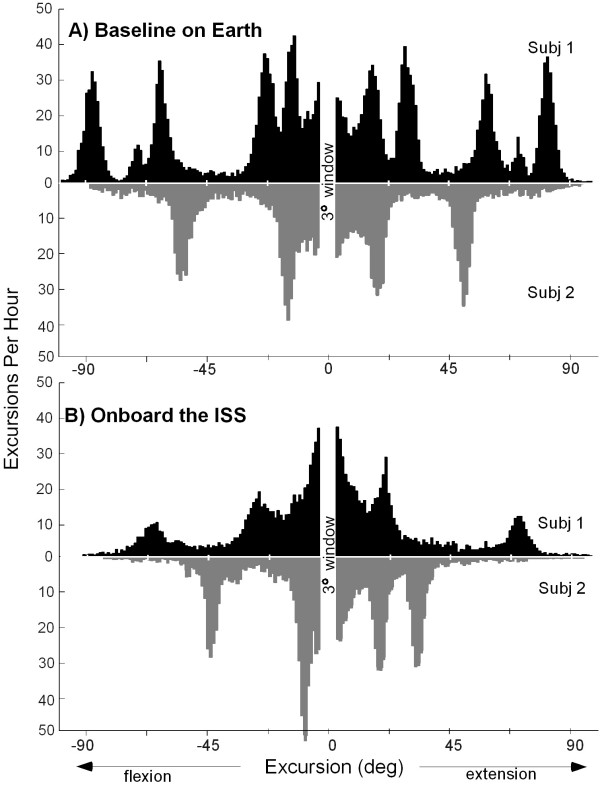
Typical histograms of the knee excursion angles (>3 °). Both subjects are shown for typical days of activity (A) on Earth and (B) onboard the ISS.

Figure 3 shows typical histograms of the excursions of the knee for different days of continuous data collection on Earth and in space. Onboard the ISS, 80.1 ± 7.4 % of all the excursions were less than 45° in magnitude compared with 55.5 ± 5.1% on Earth. There were 44% fewer excursions per hour onboard the ISS than on Earth (1320 ± 190 vs. 2360 ± 500, respectively). The sum of angles that the knee swept through per hour was 63% smaller in space (3.45 × 10^4^ ± 0.63 × 10^4 ^[in degrees] onboard the ISS vs. 9.43 × 10^4^ ± 2.2 × 10^4 ^[in degrees] on Earth).

**Figure 4:**
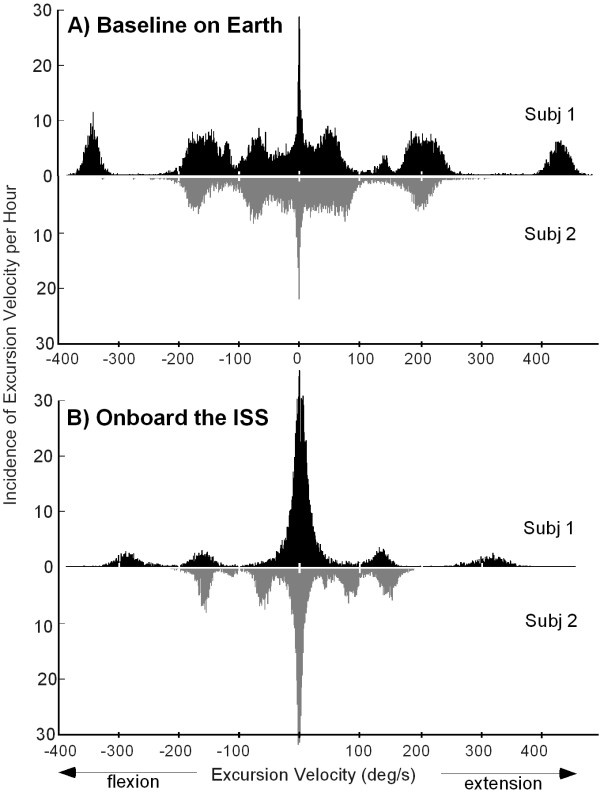
Typical histograms of the excursion velocities. The instances of different excursion velocities are shown for typical days of activity (A) on Earth and (B) onboard the ISS.

A histogram of the velocity of the excursions is shown in Figure 4. Onboard the ISS, 47.7 ± 7.6% of all the excursions occurred at velocities of <20°/s in magnitude, whereas, on Earth, only 11.8 ± 3.2% of the excursions were of <20°/s in magnitude.

### Muscle activity

**Figure 5:**
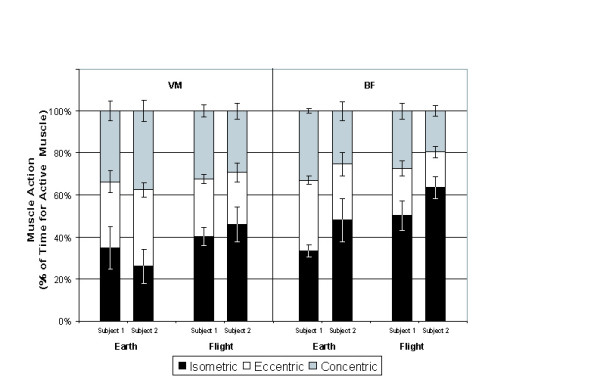
The proportion of total muscle activity in a working day that was isometric, eccentric, or concentric. Both subjects and muscles are shown for activity on Earth and onboard the ISS. Error bars indicate +/- 1 standard deviation (SD). Each set of error bars is associated with the box containing the -1 SD bar.

**Figure 6:**
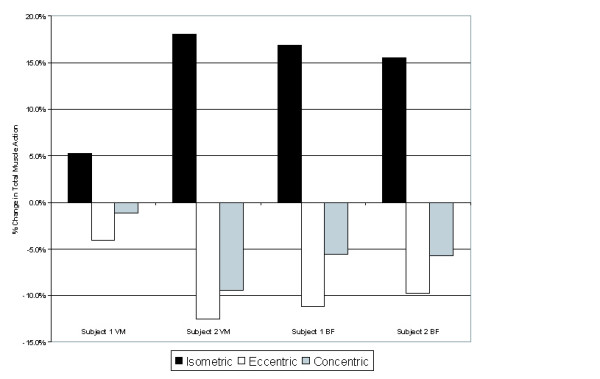
Change in the percentage duration of total muscle action onboard the ISS relative to the total muscle action duration on Earth. Mean data are presented for both subjects and both muscles. The action is either concentric, eccentric, or isometric. A positive value indicates a greater amount of that quantity onboard the ISS. Note the increase in the relative amount of isometric action for all subjects and both muscles, primarily at the expense of eccentric action.

The relative amounts of concentric, eccentric, and isometric muscle action during days on Earth and on the ISS are shown in Figure 5, and the changes in the type of muscle action in space compared with Earth are shown in Figure 6. On average, there was 5.5% less concentric muscle action, 9.4% less eccentric action, and 13.9% more isometric muscle action in space than on Earth. Overall muscle activity of the VM decreased onboard the ISS (14.2% onboard the ISS vs. 22.1% on Earth) for Subject 1 but increased for Subject 2 (25.6% onboard the ISS vs. 20.9% on Earth). Overall muscle activity of the BF increased onboard the ISS for both subjects (33.4% vs. 23.0% and 43.3% vs. 36.3% onboard the ISS vs. on Earth for Subjects 1 and 2, respectively).

## Discussion

On Earth, the angular position of the knee joint is predominantly at the two ends of the used range of motion, with the knee either flexed or extended (Figure 2A), stretching either the knee extensors or the flexors. Onboard the ISS, the knee typically is maintained at an intermediate angular position around the average modal value of 30.5 ± 13.5° (Figure 2B). The mean knee-joint angle of 41.4 ± 9.4° observed onboard the ISS was not significantly different (*p *< 0.05) from the "natural" microgravity position of 47 ± 8° reported in the NASA Standards 3000 [13]. The implications of these differences are that single knee-joint flexor and extensor muscles are not stretched to the same lengths onboard the ISS as they are on Earth. Statements regarding the length of the two-joint knee flexors and extensors would require the incorporation of both knee and hip angles into an anatomical model [11], which is beyond the scope of this report. However, given the maintained knee flexion, the flexed hip posture (which has been commonly observed in space [13]) would tend to equalize the lengths of the two-joint muscles toward their lengths during upright posture on Earth.

The "natural" position of the knee experienced in space most likely arises from the passive elastic properties of the lower-extremity joints. Extensive research has examined the passive properties of the knee joint by measuring the moment produced when the knee joint is at different angular positions throughout its range of motion [15-20]. It has been shown that the passive knee moment as a function of knee-joint angle is sigmoid in shape and that the magnitude of the moment increases exponentially as the angular position of the knee is further from a "neutral" central position. When the knee-joint angle deviates from the neutral position, passive restorative moments are produced from the imbalance in the elastic stiffness of the knee flexors and extensors. The passive properties of the hip are also likely to be critical to the "natural" posture observed.

### Reduction in knee excursions

Our data show that the total motion of the knee joint in space is greatly reduced from what is typically experienced on Earth. The data indicate both that a fewer number of excursions occurred while subjects were onboard the ISS, 44% fewer per hour, and that excursions were of smaller magnitude than on Earth. Overall, the knee was moved through a reduced span of angles; the range of motion was 63% smaller onboard the ISS than on Earth.

Peaks in the histogram of excursion angles indicate a large number of repeated motions, for instance, during walking or running (Figure 3). On Earth, both subjects exhibited such peaks at varying magnitudes covering nearly the entire range of angles used. Onboard the ISS, the amplitudes of knee movements were limited and were predominantly small, with 80.1 ± 7.4% of the excursions <45°. During activities onboard the ISS, the crewmembers made fewer and smaller-amplitude movements, resulting in less change in the angular position of the knee joint. Onboard the ISS, the knee-joint velocities indicated predominantly slower movements than those on Earth. Velocities close to zero result in quasi-isometric movements of relatively low power [21]. The maintenance of the knee joint in a flexed position during spaceflight may result in a similar flexor bias in the estimation of joint position, one that has been observed in the elbow joint [22].

### Muscle activity and action

The increased duration of muscle activity observed on the ISS for all but one of the four subject/muscle conditions studied has a precedent in the work of Edgerton and colleagues [23], who found that, compared with pre- and post-flight values, there was a marked increase in the daily integrated EMG activity of the tibialis anterior and soleus during spaceflight. However, the analysis presented here refers only to the duration of above-threshold activity, and thus the magnitude of activity (which was incorporated into the Edgerton group's data) is not considered. This information is available and will be the topic of future communications. The change to a more dominant pattern of isometric action onboard the ISS is reasonable based on the anti-gravity role of the muscles studied on Earth. The marked reduction in knee-joint velocities observed in space suggests a change in the pattern of muscle use, which is likely to be associated with the change in expression of the myosin phenotypes that has been observed from human biopsy studies [7,24].

Among the limitations of the current experiment are the potential for migration and/or misalignment of the goniometers and the simplicity of the muscle-length models. There is also a possibility that the electrode-skin interface changed during the approximately 8-hour data collection sessions, although we have previously shown that EMG in response to a standard load measured on multiple occasions over the course of a day in which the electrodes are not removed is highly reliable [25]. However, all of the above factors could have exerted an influence during experiments on Earth or in space, and thus no bias in the results is likely.

## Conclusion

Onboard the ISS, the knee is operated in different ranges of angles, excursions, total daily excursion, and velocities than those observed during typical daily activity on Earth. These differences imply that the muscles spanning the knee joint are operating at altered lengths, velocities, and power ranges, all of which may contribute to the muscle atrophy and functional losses that have been observed in microgravity.

## Abbreviations

BF – Biceps Femoris

EMG – Electromyography

ISS – International Space Station

LEMS – Lower Extremity Monitoring Suit

RMS – Root Mean Square

SD – Standard Deviation

VM – Vastus Medialis

## Competing interests

The author(s) declare that they have no competing interests.

## Authors' contributions

MCP contributed to data analysis, interpretation of data, and drafting the manuscript. KOG contributed to data analysis and drafting the manuscript. ML and BH were responsible for developing the data analysis algorithms and assisted with manuscript revisions. AJR was responsible for project organization and contributed to data acquisition and manuscript revision. CCM managed the in-flight aspects of data collection. PRC conceived and designed the experiment, contributed text, and critically reviewed the manuscript. All authors have read and approved the final manuscript.
